# Dental Rehabilitation for Free Fibula Flap-Reconstructed Mandible with Scar Contracture: A Technical Note

**DOI:** 10.3390/dj7030065

**Published:** 2019-06-29

**Authors:** Masaya Akashi, Kousuke Matsumoto, Daisuke Takeda, Junya Yamashita, Nanae Yatagai, Kazunobu Hashikawa, Takahide Komori

**Affiliations:** 1 Department of Oral and Maxillofacial Surgery, Kobe University Graduate School of Medicine, Kobe 650-0017, Japan; 2 Department of Oral and Maxillofacial Surgery, Kobe Central Hospital, Kobe 650-0017, Japan; 3 Department of Plastic Surgery, Kobe University Graduate School of Medicine, Kobe 650-0017, Japan

**Keywords:** vestibuloplasty, polyglycolic acid sheet, skin graft, free fibula flap, custom titanium bar

## Abstract

Dental rehabilitation with osseointegrated implants in reconstructed mandibles is a common procedure, but the technique still requires improvement, especially in its reliability and technical simplification. We herein report dental rehabilitation of a free fibula-reconstructed mandible with scar contracture. A vestibuloplasty technique with application of a polyglycolic acid (PGA) sheet is described. The implants were inserted into a viable fibula flap with severe scar contracture of the overlying epithelium resulting from vascular instability in skin paddle. Only the fibula periosteum was sutured after implant insertion; exposed surfaces were covered with a combination of PGA sheet and fibrin sealant. The area with PGA sheet coverage gradually healed with moderate contracture. The epithelium around the almost implants became immobilized. The implant-supported removable partial denture with custom titanium bar was acceptable. Dental rehabilitation is possible for reconstructed mandibles with severe scar contracture. Application of a PGA sheet may be useful for vestibuloplasty in patients with reconstructed mandibles.

## 1. Introduction

Dental rehabilitation following jaw resection is one of the most challenging and worthwhile procedures for oral and maxillofacial reconstructive surgeons. Composite free flap transfer is a common choice for jawbone defects. The fibula flap has long been one of the predominate bone flaps for jaw reconstruction [1]. Osseointegrated implant-supported prosthesis is preferable for functional and esthetic restoration [2,3].

Implant surface modifications have an important role for the success of osseointegration [4]. A previous cadaveric study revealed that the fibula bone has adequate width, thickness, and bone volume for dental implant placement that can withstand the biomechanical loads of mastication forces experienced throughout a lifetime [5]. In fact, a recent retrospective study reported that the success rate of implants placed in fibula flaps was 92% with an average follow-up of 30 months [1]. The condition of perimplant soft tissue is critical for long-term marginal bone stability around dental implants, especially in reconstructed jaws [6]. Extraoral skin paddles of the fibula flaps, non-attached alveolar mucosa, and missing vestibule lead to poor hygiene around dental implants, resulting in chronic inflammation followed by peri-implant marginal bone loss [6]. Reconstructed skin seems not to be a suitable tissue for use around implants [2].

Various techniques have been reported to promote the formation of attached mucosa around dental implants [7]. A palatal mucosal graft after skin removal may be the best option to gain an adequate zone of firmly attached mucosa for the relatively small-sized wound [2,8]. Xenogenic graft materials are known to be sufficient for soft tissue augmentation to increase keratinized mucosa in implant surgery [9]. Although a split-thickness skin graft (STSG) is accepted for vestibuloplasty, particularly in patients undergoing intraoral osteocutaneous reconstruction with a free flap, the constant mobility of the tongue and cheeks and saliva secretion can cause unfavorable healing [10]. Bare bone grafts with vascularized iliac crest have been used for mandibular reconstruction; these grafts acquire attached mucosa from healing by secondary intention without suturing of a skin paddle in the oral cavity [7].

Polyglycolic acid (PGA) is an absorbable suture-reinforcement material [11]. The usefulness of covering partial glossectomy wounds with a PGA sheet and fibrin glue spray was recently reported [11,12]. Herein we describe dental rehabilitation using vestibuloplasty with a PGA sheet and custom titanium bar with locator abutments for a free fibula flap-reconstructed mandible with severe scar contracture.

## 2. Case Presentation

**Figure 1:**
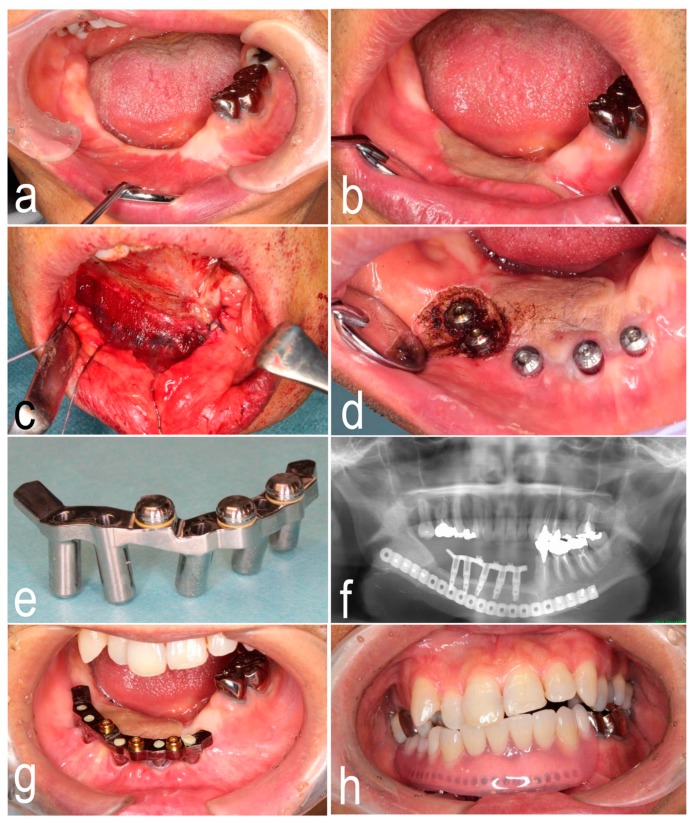
(**a**) Narrow oral vestibular area with severe scar contracture. (**b**) Partially engrafted skin graft on the lingual side. (**c**) Polyglycolic acid sheet covering after closure of fibular periosteum. (**d**) Immobilized epithelium around the healing abutments in the anterior mandibular region and laser ablation in the posterior mandibular region. (**e**) Custom titanium bar with locator abutments. (**f**) Panoramic X-ray image showing placement of titanium bar. (**g**) Placement of titanium bar with locator abutments. (**h**) Implant-supported removable partial denture.

A 55-year-old man underwent ablative surgery for sclerosing odontogenic carcinoma and simultaneous reconstruction with a free fibula osteocutaneous flap. Written informed consent was obtained from the patient for the publication of the case report and the use of clinical photographs. Vascular instability in the skin paddle alone was found after surgery. Although healing by secondary intention attributed to careful and gradual debridement of skin paddle was achieved on the viable fibula bone flap (Figure 1a), the narrow and mobile oral vestibular area resulted in denture instability postoperatively. Two years after the cancer surgery, osteosynthesis fixation screw removal by extraoral approach and intraoral vestibuloplasty with STSG was performed for osseointegrated implant insertion. The STSG was partially engrafted, but the gain of attached mucosa, especially on the labial side, remained insufficient (Figure 1b). An additional vestibuloplasty on the labial side was necessary, but the patient did not want to receive a skin graft again.

Three months after osteosynthesis fixation screw removal and STSG, implant insertion was performed in the fibula bone. The boundary between the oral mucosa and the engrafted skin was sharply incised. The implants were inserted with a surgical stent at the positions determined with preoperative simulation. Fresh bleeding was observed from the drilled holes, confirming fibula flap viability. We predicted that the problematic vertical and horizontal gaps between the fibula bone and the overlying epithelium would remain postoperatively. Only the fibula bone periosteum was sutured. First, a small amount of fibrin sealant (Beriplast P; CSL Behring Gmbh, Marburg, Germany) was rubbed onto the exposed surfaces over the periosteum, and then a PGA sheet (Neoveil; Gunze Co., Ltd., Tokyo, Japan) was affixed to the wound. Finally, the fibrin sealant was sprayed on with a special spray kit (Figure 1c). Intra- and extraoral fixation with suturing was not performed. Five days after implant insertion, the patient resumed wearing a removable partial denture. A hard chairside denture reline resin was added on the labial side of the denture to maintain space in the labial vestibule, and such denture adjustment was continued according to the wound condition after the first week after surgery. The patient was instructed to perform lower-lip extension exercises to prevent severe scar contracture. The area with PGA sheet coverage gradually healed with moderate contracture. The soft tissue around the healing abutments in the anterior mandibular region was adequately immobilized (Figure 1d).

Inflammatory granulation tissue around the healing cap in the molar region was ablated with a laser as needed (Figure 1d). The epithelium around the implants in the molar region gradually became immobilized with repeated laser ablation of granulation tissue. To compensate for the residual vertical gap between the fibula bone and the overlying epithelium, a custom titanium bar with locator abutments was manufactured and placed 6 months after implant insertion (Figure 1e). The patient was satisfied with the stability and function of the implant-supported removable partial denture (Figure 1f–h). Eighteen months after implant placement, no hyperplastic/inflammatory response and formation of the granulomatous tissue around implants were found.

## 3. Discussion

It remains to be elucidated whether it is preferable to insert dental implants into a skin flap or attached epithelium acquired with STSG or gingival grafts [13,14]. As shown in the current report, in patients with severe scar contracture of overlying epithelium, retention of removal partial dentures is poor and soft tissue management around the implant is difficult. Although the STSG of the vestibuloplasty was partially engrafted, the expansion on the labial side was insufficient because of the abnormal relationship between the maxilla and reconstructed mandible and the vertical thickness between the fibula bone and overlying scar tissue.

We applied a PGA sheet after additional vestibuloplasty on the labial side. A PGA sheet is useful for vestibuloplasty in reconstructed mandibles because as a xenomaterial it probably inhibits rapid epithelization that results in severe scar contracture. The basis of this hypothesis relies on a previous report by Kumar et al. [15] in which a denture was used to restrict excessive proliferation of regenerating epithelial tissue and reliably produce epithelium of adequate thickness and consistency attached to the fibula. We applied a similar method by using a partial denture. In the method of Kumar et al., the tip of the dissected skin paddle is repositioned to form the buccal/labial vestibule and the floor of the mouth on the lingual side and fixed with suturing into the underlying tissues or with transcutaneous sutures [15]. We performed vestibuloplasty with STSG and anchored the tips of grafted skin with transcutaneous sutures before implant insertion. In detail, the intraoral suture was initially placed by inserting into the labial edge of wound and then pulled out transcutaneously. Extraoral fixation was done by using a catheter at the skin near the submental area (see Figure S1 in the Supplementary Materials). However, the expansion of attached epithelium on the labial side was insufficient. Therefore, a second vestibuloplasty was necessary.

PGA acts as a protective barrier until wound closure [16]. It is hypothesized that covering defects with protective barriers reduces inflammation and suppresses the excessive proliferation and collagen secretion that result in unfavorable scar contracture. PGA is known to reduce the expression of α-smooth muscle actin (marker of myofibroblasts, the shrinking of which causes scar contracture). Importantly, Kibe et al. [16] reported that there was little invasion of epithelial cells into PGA, whereas epithelial cells multiplied on the surface of granulation tissue in open wounds. In an animal experiment, Inokuchi et al. [17] showed that the area of scar tissue in a group without any graft was deeper and narrower than that in a group with PGA-covered wounds. This finding indicates that a PGA covering is likely to induce scar formation with a wide surface area, which leads to moderate contracture. Although some believe that application of a PGA sheet and fibrin glue spray to bone surfaces during oral surgery promotes epithelization [18], it seems that, as a xenomaterial, PGA is suitable for controlling the size and speed of wound epithelization by suppressing rapid invasion of epithelial cells rather than promoting epithelization.

The advantage of PGA sheet application is the non-invasiveness, but there are some disadvantages as follows. Although the periosteum of the fibula bone was sutured in our method, there are still potential risks of bacterial contamination and implant infection. The risk of developing granuloma-like neoplasm with the application of PGA sheets and fibrin glue following resection of oral mucosa was recently reported [19]. Appropriate treatments are necessary, such as laser ablation of granulation tissue around the inserted implants. Repeated laser ablation may promote scar formation, resulting in gain of immobilized mucosa around implants. If peri-implant soft tissue management is not sufficient with repeated laser treatment, a palatal mucosal graft may be necessary to obtain the keratinized mucosa. When acceptable attachment of mucosa around the implants is acquired but a vertical gap remains between the fibula and epithelium, the application of a custom titanium bar is useful.

## 4. Conclusions

Dental rehabilitation of a fibula free flap-reconstructed mandible with sever scar contracture of overlying epithelium is difficult but possible. Application of a PGA sheet can be useful for vestibuloplasty in reconstructed mandibles.

## Supplementary Materials

The following are available online at https://www.mdpi.com/2304-6767/7/3/65/s1. Figure S1: Primary vestibuloplasty.
