# Supplementary figures and images for: Dental Rehabilitation for Free Fibula Flap-Reconstructed Mandible with Scar Contracture: A Technical Note
Source: Dent J (Basel). 2019 Jun 29;7(3):65. doi: 10.3390/dj7030065 (PMC6784466; doi:10.3390/dj7030065)

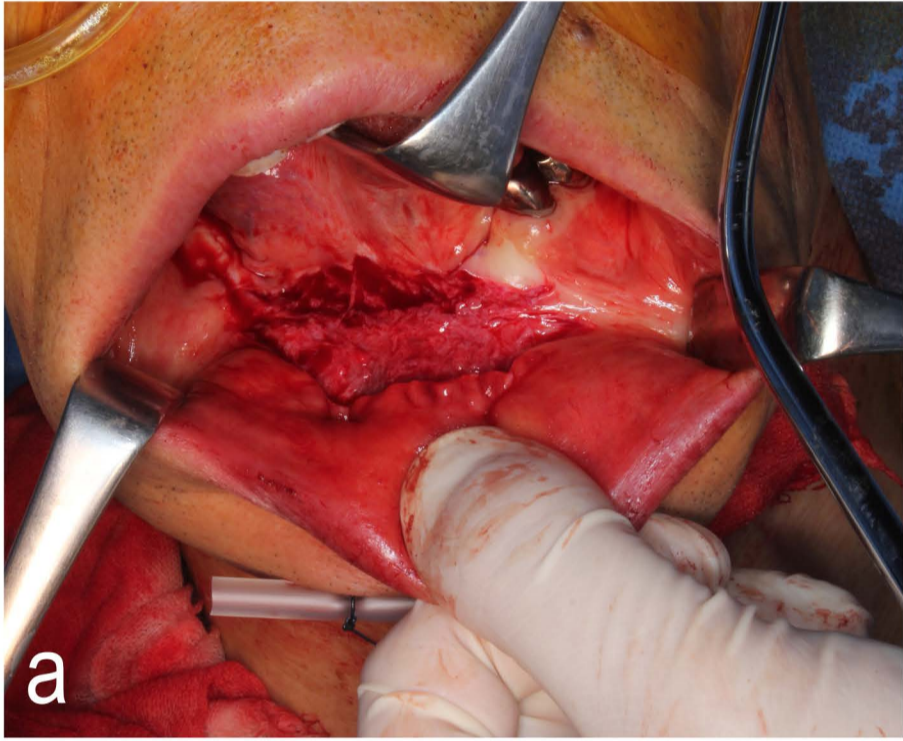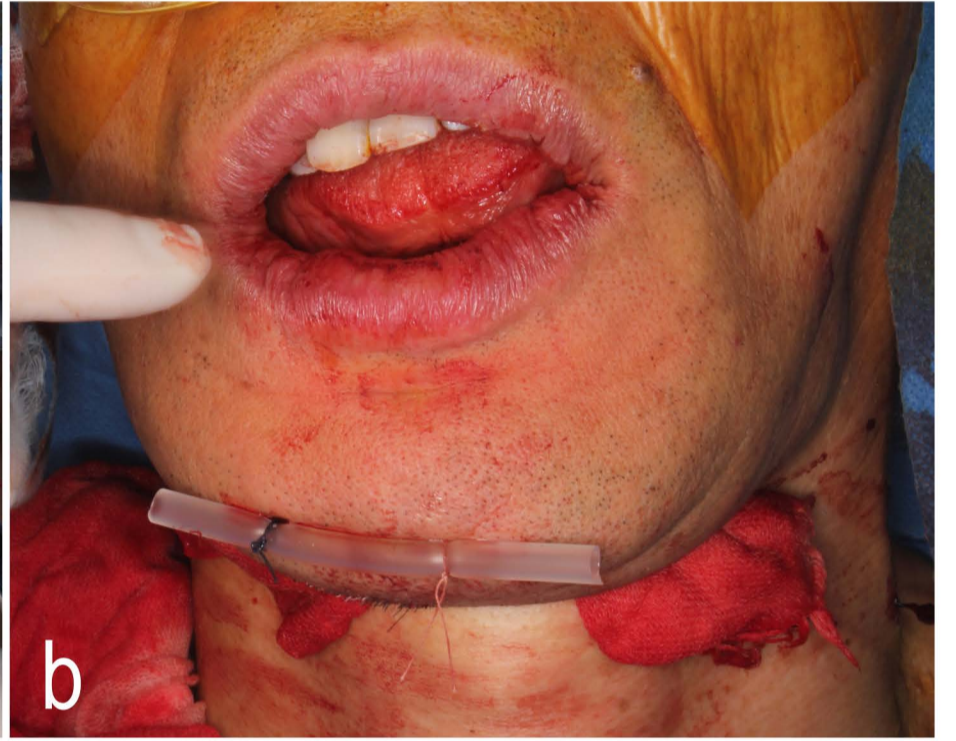

**Supplemental figure**

Supplement: Supplementary file 1 [file dentistry-07-00065-s001.pdf]
